# Outcomes for recurrent or metastatic head and neck cancer by HPV status: a systematic review and meta-analysis

**DOI:** 10.1093/oncolo/oyaf043

**Published:** 2025-04-11

**Authors:** Jong Chul Park, Geun Hee Ye, Ross Merkin, Thomas Roberts

**Affiliations:** Department of Medicine, Massachusetts General Hospital, Boston, MA 02114, United States; Department of Medicine, Harvard Medical School, Boston, MA 02115, United States; Department of Medicine, CHA University School of Medicine, Pocheon, Gyeonggi-do, 11160, South Korea; Department of Medicine, Massachusetts General Hospital, Boston, MA 02114, United States; Department of Medicine, Harvard Medical School, Boston, MA 02115, United States; Department of Medicine, Massachusetts General Hospital, Boston, MA 02114, United States; Department of Medicine, Harvard Medical School, Boston, MA 02115, United States

**Keywords:** human papillomavirus, head and neck cancer, survival outcome

## Abstract

**Background:**

Human papillomavirus (HPV)-positive (HPV+) recurrent or metastatic head and neck squamous cell carcinoma (R/M HNSCC) exhibits distinct clinical behavior compared with HPV-negative (HPV−) R/M HNSCC. However, most studies reference historical efficacy benchmarks from cohorts combining HPV+ and HPV− R/M HNSCC for comparison with the efficacy of novel therapeutics. This analysis aims to assess differential outcomes specific to HPV+ and HPV− R/M HNSCC to reflect HPV-associated prognostic considerations.

**Methods:**

Studies from March 2006 to June 2024 were searched. All therapeutic trials in R/M HNSCC that reported outcomes by HPV status were included. Meta-analyses were conducted to evaluate overall survival (OS) as the primary outcome of interest, objective response rate, and progression-free survival as secondary outcomes. Weighted averages were used to estimate the outcome measures for each group.

**Results:**

A total of 10 studies representing 3509 patients met the inclusion criteria. For the first-line setting, the weighted average OS estimates were 20.7 months (95% CI, 19.4-21.9) and 12.2 months (95% CI, 11.9-12.4) for HPV+ and HPV− HNSCC, respectively. For the second or later line treatment setting, the weighted average OS estimates were 11.1 months (95% CI, 10.2-12.1) and 8.3 months (95% CI, 7.9-8.6) for HPV+ and HPV− subgroups, respectively.

**Conclusion:**

Our analysis demonstrated a clinically meaningful difference between in the HPV+ and HPV− R/M HNSCC populations. These findings support the establishment of HPV-specific outcome benchmarks, which are essential for prognostic awareness, optimizing therapeutic development, and accurately interpreting clinical trial data.

**Implications for Practice**

The improved survival outcomes observed in HPV-positive R/M HNSCC are consistent across the majority of recent therapeutic trials and highlight the distinct biology of HPV-positive versus HPV-negative HNSCC. These findings are important for prognostication and should be considered in clinical trial design and therapeutic strategies.

## Introduction

The rising incidence of HPV-positive HNSCC has significantly shifted its epidemiology in recent decades, yet HPV-negative cases, mainly caused by smoking, remain more frequent in Western countries despite declining tobacco consumption.^1^ HPV + HNSCC primarily affects the oropharynx and is driven by high-risk HPV genotypes, predominantly HPV16. Oncogenesis in these cases is mediated predominantly by viral oncoproteins E6 and E7, which disrupt tumor suppressor pathways.^2^

Patients with localized or locally advanced HPV + HNSCC exhibit high response rates to radiotherapy and chemotherapy, resulting in a better prognosis compared with HPV− HNSCC.^3^ Acknowledging the distinct prognosis and clinical behavior of HPV+ oropharyngeal cancer (OPC), the eighth edition of the American Joint Committee on Cancer staging system introduced a separate clinical and pathological TNM classification for HPV + OPC. This change reflects the unique behavior and better prognosis of HPV + OPC compared with HPV− OPC. The improved responsiveness of HPV + OPC to radiotherapy and chemoradiotherapy has sparked ongoing efforts to de-escalate treatment intensity for HPV + HNSCC patients, such as reducing radiation doses or modifying chemotherapy regimens. These efforts aim to reduce toxicity without compromising oncological outcomes, with varying degrees of success. In contrast to localized disease, the standard-of-care treatment options for patients with R/M HNSCC do not vary by HPV status. Overall, systemic therapy options for R/M HNSCC are limited to single agents or combinations of immune checkpoint inhibitors (ICI), cetuximab, and chemotherapy. No new therapies have been approved by the FDA for R/M HNSCC since the approval of anti-PD1 ICIs, such as pembrolizumab and nivolumab, in 2016.^4^ Additionally, cetuximab is considered less effective in HPV + HNSCC compared with HPV− HNSCC, further limiting treatment options.^5,6^ Recently, a variety of HPV-targeting novel therapeutics have entered clinical trials for patients with HPV + R/M HNSCC and have demonstrated promising efficacy in single-arm studies.

However, there is still no consensus on the prognostic and treatment response benchmarks specific to the HPV+ and HPV− R/M HNSCC populations. KEYNOTE-048, the trial that defines the current first-line treatment for most patients with HNSCC and is commonly used as a historical benchmark for emerging therapeutics, did not report OS or progression-free survival (PFS) by HPV status. Additionally, many post-ICI treatment decisions rely on extrapolation from older clinical trials that were published before the prognostic value of HPV in HNSCC was fully understood. As a result, current research often reports efficacy benchmarks derived from studies that include mixed populations of HPV+ and HPV− patients.^4^ Given the differences in underlying biology and prognosis based on HPV status, defining clear efficacy benchmarks for R/M HNSCC stratified by HPV status is essential to appropriately evaluate novel therapeutics that select patients based on HPV status. This systematic review and meta-analysis aims to assess oncologic outcomes based on HPV status and support the need to establish consensus benchmarks for clinical trial design.

## Methods

### Inclusion and exclusion criteria

Our selection criteria were established using the PICOS framework (Population, Intervention, Comparison, Outcome, and Study Design). We included clinical trials that prospectively evaluated therapies in patients with R/M HNSCC. Studies were considered eligible if they provided subgroup databased on HPV status for patients with R/M HNSCC. We included studies that validated HPV status by either immunohistochemistry for p16 expression, HPV in-situ hybridization, or polymerase chain reaction for detecting HPV DNA.

Studies were excluded based on the following criteria: (1) studies focused on conditions other than R/M HNSCC, (2) studies that did not report results specific to HPV+ or HPV− R/M HNSCC patients, (3) studies that did not provide data on OS, (4) studies presenting duplicated data previously published, (5) case reports, case series, observational studies, preclinical research, retrospective analyses, and (6) studies not written in English.

### Information sources and search strategy

We conducted a systematic literature search across PubMed, Cochrane, EMBASE, SCOPUS, Web of Science, ClinicalTrials.gov, major oncology conference publications and abstracts, Google Scholar, and ProQuest using the following search terms: HPV, recurrent, metastatic, head and neck squamous cell carcinoma, and treatment. The search included all articles published up to June 2024.

### Study selection and data collection process

Two researchers (J.P. and G.Y.) independently screened the titles and then abstracts of all references identified. Subsequently, the same researchers independently assessed the full-text articles of these selected studies, applying the inclusion criteria. Any discordances were resolved through discussion. Multiple publications from the same trial (identified by the same NCT number) were treated as one study, using the most recent publication as the primary source for outcome data.

Two authors (J.P. and G.Y.) independently extracted key data from the selected studies. Information collected included report details, study characteristics, patient population details, interventions, results, and main conclusions. Extracted data were compared, and discrepancies were resolved through discussion. GY entered the data into RevMan Web Version 5.4. The Cochrane Collaboration, 2020.

### Data analysis

We evaluated the risk of bias in the included studies using the revised Cochrane “Risk of Bias” tool for randomized trials (RoB 2) via RevMan Web Version 5.4. Two authors (J.P. and G.Y.) independently applied the tool, documenting justifications for each domain’s risk of biased judgment. Discrepancies were resolved through discussion.

The primary analysis focused on OS stratified by HPV status. Secondary outcomes included objective response rate (ORR) and PFS by HPV status. We used the Median Survival Ratio (MSR) as a summary statistic to compare the efficacy of treatments between HPV+ and HPV− groups, following the method described by Hirst et al. (2021).^7^

Random effects meta-analyses were conducted using RevMan Web Version 5.4. (The Cochrane Collaboration, 2020). Weighted averages for OS, PFS, and aggregated ORR were calculated using Microsoft Excel 2021 (Microsoft Corporation). All results are presented with 95% CIs. For detailed synthesis methods, see Supplementary Material.

### Protocol and registration

This systematic review follows the Preferred Reporting Items for Systematic Reviews and Meta-Analyses (PRISMA) 2020 guidelines. The review protocol is registered in the Prospective Register of Systematic Reviews (PROSPERO) with registration number CRD42024559638.

## Results

### Study selection

**Figure 1.**
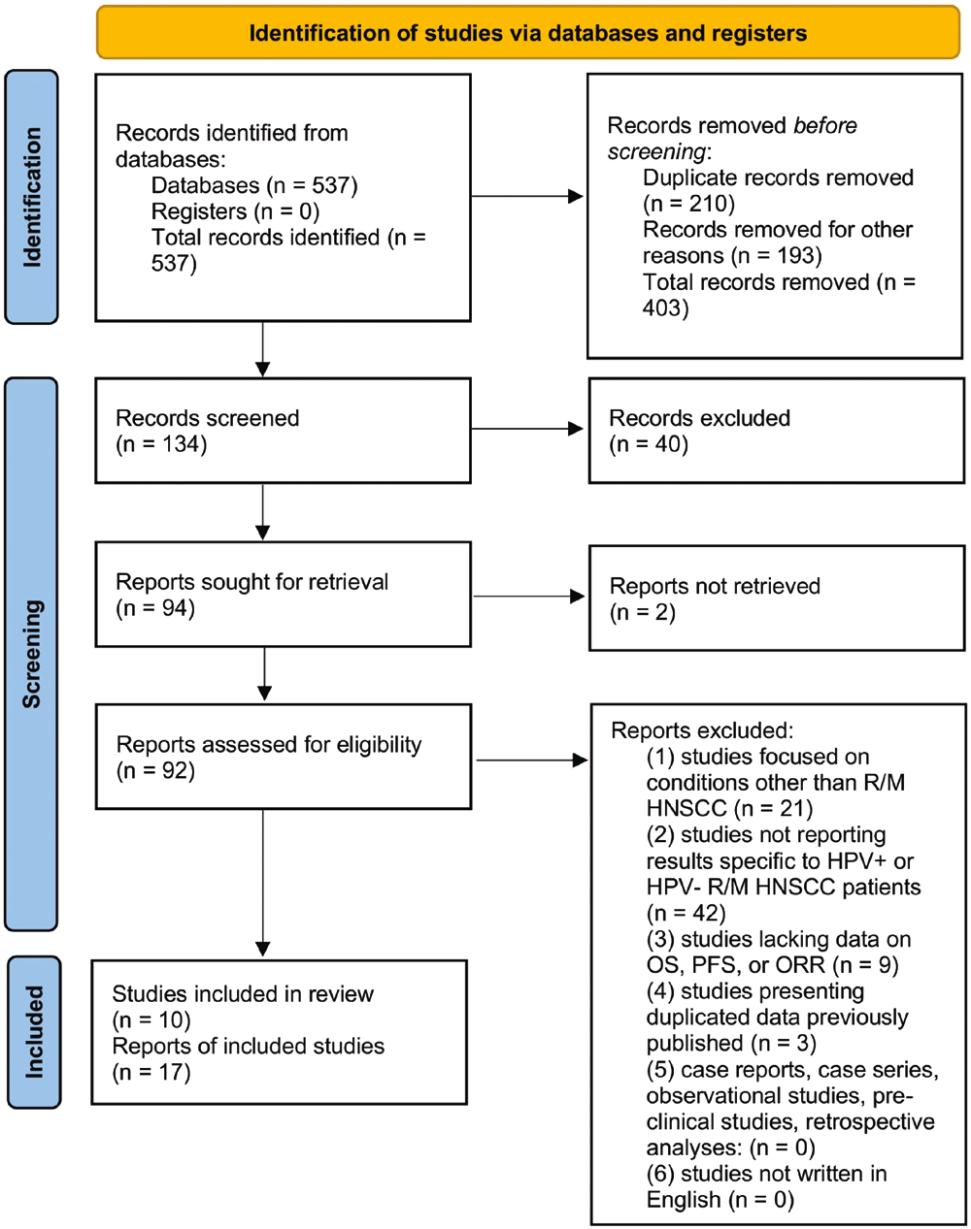
Flow diagram of literature search and selection criteria adapted from PRISMA. Abbreviations: HPV, human papillomavirus; OS, overall survival; ORR, objective response rate; PFS, progression-free survival; R/M HNSCC, recurrent or metastatic head and neck squamous cell carcinoma.

From the 537 records identified, we reviewed 92 full-text documents and identified 10 studies that met our inclusion criteria (Figure 1). These studies are listed in Table 1. Furthermore, searches of the initially included studies yielded no additional articles meeting our inclusion criteria. Most studies had a low overall risk of bias (Supplementary Figure 1).

**Table 1.** Summary of efficacy outcomes of the first-line and second or later-line studies in R/M HNSCC by HPV status.

	ID/Trial Number	Year	Study design	Intervention	HPV status	*N*	mOS (m)	95% CI	ORR (%)	95% CI	mPFS (m)	95% CI
**First-line**	GORTEC 2014-01 TPExtreme/NCT02268695^8^	2021	Randomized active-controlled phase II	TPExtreme (docetaxel + cisplatin + cetuximab)	HPV+	20	36.2	15.2-NR			6.3	4.5-21.6
					HPV−	84	14.5	11.1-16.9			6.2	5.5-7.9
				EXTREME (fluorouracil + cisplatin + cetuximab)	HPV+	14	21.1	9.6-NR			6.2	4.4-12.7
					HPV−	62	11.3	8.6-15.2			6.1	4.7-7.7
	CheckMate 651 / NCT02741570^9^	2023	Randomized active-controlled phase III	Nivolumab + ipilimumab	HPV+	186	19.8					
					HPV−	761	13.1					
				EXTREME	HPV+	186	23.8					
					HPV−	761	12.6					
	SPECTRUM / NCT00460265^10^	2013	Randomized phase III	Cisplatin + fluorouracil + panitumumab	HPV+	57	11.0	7.3-12.9			5.6	4.4-6.5
					HPV−	179	11.7	9.7-13.7			6.0	5.6-6.9
				Cisplatin + fluorouracil	HPV+	42	12.6	7.7-17.4			5.5	3.4-6.7
					HPV−	165	8.6	6.9-11.1			5.1	4.1-5.5
	CheckMate 714 / NCT02823574^11^	2023	Randomized phase II	Nivolumab + ipilimumab (platinum-eligible)	HPV+	35	18.6	8.5-28.1				
					HPV−	88	7.8	5.1-12.4				
				Nivolumab (platinum-eligible)	HPV+	17	33.7	12.9-NR				
					HPV−	44	9.5	5.3-14.0				
**Second or later-line**	CheckMate 141 / NCT02105636^12^	2016	Randomized active-controlled phase III	Nivolumab	HPV+	64	9.1	6.5-11.8	15.9			
					HPV−	56	7.7	4.8-13.0	8			
				Investigator’s choice	HPV+	29	4.4	3.0-9.8	3.4			
					HPV−	37	6.5	3.9-8.7	11.1			
	HAWK / NCT02207530^13^	2019	Single-arm phase II	Durvalumab	HPV+	34	10.2	7.2-16.3	29.4	15.1-47.5	3.6	1.9-5.6
					HPV−	65	5.0	3.4-8.4	10.8	4.4-20.9	1.8	1.6-2.0
	JAVELIN Solid Tumor / NCT01772004^14^	2021	Phase Ib basket	Avelumab	HPV+	39	11.8	7.8-16.3	15.4	5.9-30.5	2.7	1.4-3.9
					HPV−	99	7.4	5.0-8.7	5.1	1.7-11.4	1.4	1.4-1.4
	KEYNOTE-012 / NCT01848834^15^	2018	Phase Ib basket	Pembrolizumab	HPV+	20	NR	8-NR	24	13-40	4.0	2-10
					HPV−	36	8.0	4-NR	16	10-23	2.0	2-4
	NCT03370276^16^	2022	Phase II	Nivolumab + cetuximab (Cohort A + B)	HPV+	48	15.3	8.0-19.9	18		4.1	2.0-7.5
					HPV−	40	12.4	9.7-14.5	41		5.6	3.1-10.8
	CheckMate 714 / NCT02823574^17^	2017	Randomized phase II	Nivolumab + ipilimumab (platinum-refractory)	HPV+	30	13.9	6.0-NR				
					HPV−	129	9.5	6.3-10.9				
				Nivolumab (platinum-refractory)	HPV+	16	14.3	6.3-NR				
					HPV−	66	9.6	6.9-13.4				

Abbreviations: HNSCC, head and neck squamous cell carcinoma; HPV, human papillomavirus; mOS, median overall survival; mPFS, median progression-free survival; *N*, number; NR, not reached; ORR, objective response rate.

### First-line treatment setting

**Figure 2.**
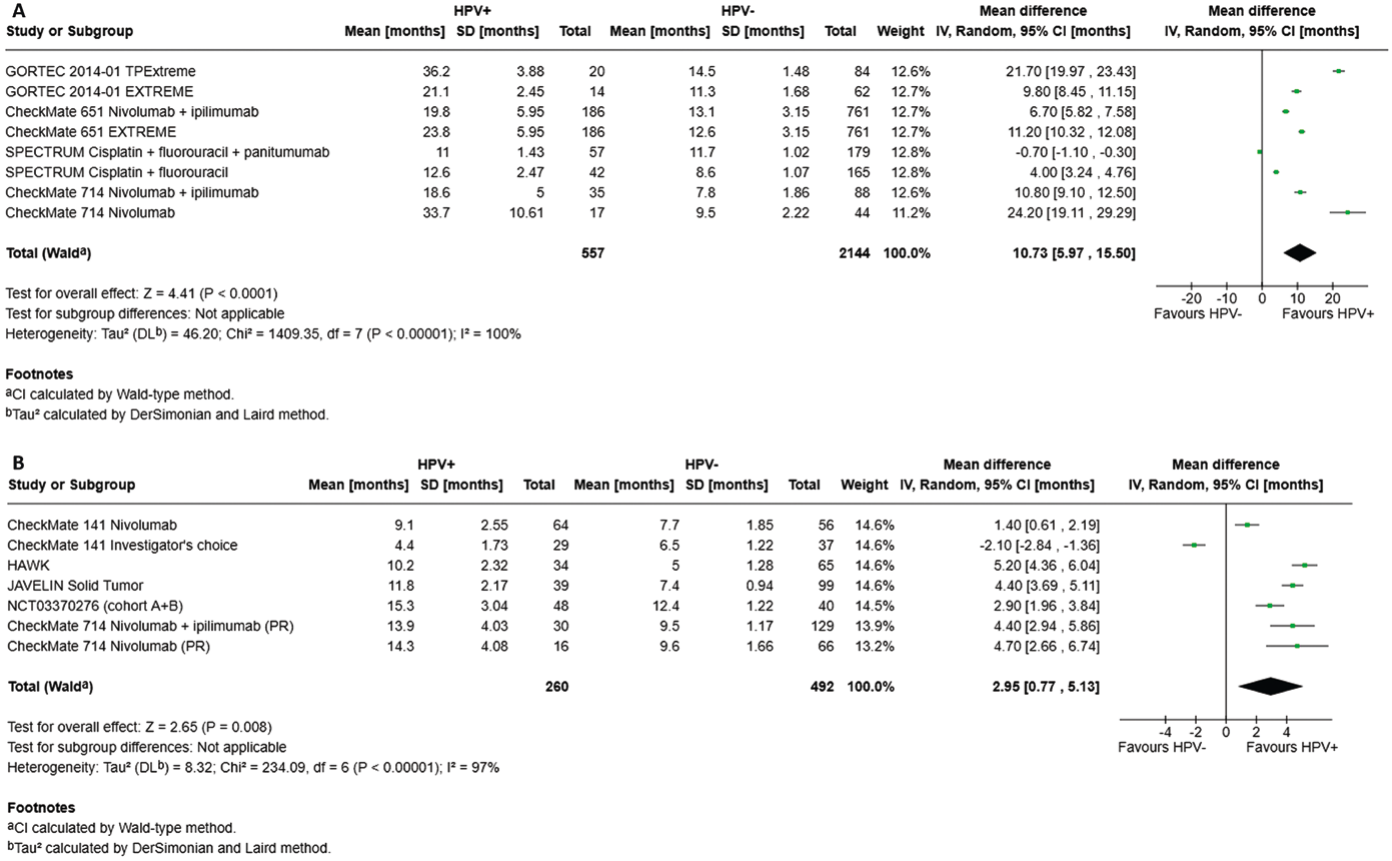
Forest plots for OS in the first-line (A) and second or later-line (B) treatment settings. Abbreviations: HPV, human papillomavirus; OS, overall survival.

Four trials reported survival data specifically for the HPV+ and HPV− subgroups in first-line R/M HNSCC patients (*n* = 2701, Table 1). A meta-analysis of the studies, which included GORTEC 2014-01 TPExtreme, CheckMate 651, SPECTRUM, and CheckMate 714, demonstrated superior OS in HPV + subgroups compared with their HPV− counterparts. In the first-line setting, the weighted average OS estimates were 20.7 months (95% CI, 19.4-21.9) and 12.2 months (95% CI, 11.9-12.4) for HPV+ and HPV− HNSCC, respectively. The pooled MSR for OS in the first-line treatment setting was 1.72 (Table 1, Figure 2A). None of the studies reported ORR values by HPV status, while PFS values by HPV status were reported in 2 studies, which were not different between subgroups (Table 1).

### Second or later-line treatment setting

Six trials investigating second or later-line therapies in R/M HNSCC patients reported survival data specifically for the HPV+ and HPV− subgroups (*n* = 808, Table 1). A meta-analysis of the studies, including CheckMate 141, HAWK, JAVELIN Solid Tumor, KEYNOTE-012, NCT03370276, and CheckMate 714, demonstrated longer survival among patients with HPV + HNSCC compared with those with HPV− disease. In the second or later-line treatment setting, the weighted average OS estimates were 11.1 months (95% CI, 10.2-12.1) and 8.3 months (95% CI, 7.9-8.6) in HPV+ and HPV− subgroups, respectively. The pooled MSR for OS in the second or later-line treatment setting was 1.10 (Table 1, Figure 2B). The PFS and ORR values for the second or later-line setting are summarized in Table 1.

## Discussion

The superior prognosis of locally advanced HPV + HNSCC compared with HPV− HNSCC is well-established. However, clinical trial designs often do not account for the expected differences in survival between HPV+ and HPV− patients, and outcome benchmarks are typically based on historical data from mixed HPV+ and HPV− populations. This study challenges that approach by demonstrating differences in overall survival (OS) based on HPV status across multiple trials where data were reported by HPV status.

Our analysis demonstrates that OS was clinically meaningfully better in the HPV+ group than in the HPV− group in both the first-line and subsequent-line treatment settings, regardless of treatment modality. Only the experimental arm of SPECTRUM, which evaluated the addition of panitumumab to first-line therapy, and the control arm of Checkmate 714, in which many patients were treated with cetuximab-containing regimens, demonstrated equivalent or superior OS in the HPV− group compared with the HPV+ group. Similarly, only NCT03370276, the phase II study of nivolumab and cetuximab (NCT03370276) and the control arm of Checkmate 714 reported higher ORRs in the HPV− group.^16^ These findings align with prior observations that the efficacy of cetuximab and other EGFR-targeted therapies is lower in HPV + HNSCC.^5^

While we recognize that the ORR and PFS outcomes are treatment regimen-dependent and cannot be universally applied, we reported these outcome measures to support our hypothesis and underscore the need for new outcome benchmarks for HPV + HNSCC. This is particularly relevant as increasing numbers of novel HPV-targeted therapeutics are being tested in HPV + HNSCC, and EGFR-targeting drug development often focuses on the HPV− population.

The primary limitation of this study is the small number of trials that met our inclusion criteria, which may affect the generalizability of our findings. Notably, although OS hazard ratios by HPV status were reported in KEYNOTE-048, which defines the current standard of care for first-line R/M HNSCC, this study was excluded from our analysis because it did not report median OS or PFS by HPV status, which are necessary for performing a meta-analysis. As the analysis is based primarily on subgroup data, we noted wide variations (CI) in OS data in some studies, particularly those with small sample sizes. We recognize that these large variations may pose challenges in applying the benchmark survival data to clinical trial design. The timepoint-specific survival rate analysis, which could provide insights into the durability of response as well as survival patterns and trends, could not be conducted due to the unavailability of adequate dataset. Additionally, heterogeneity of patient demographics, treatment modality, eligibility criteria, and prior treatment histories may have introduced sources of bias. Some studies included in our analysis were conducted during an era when p16 overexpression was considered an acceptable surrogate marker for HPV infection without requiring confirmatory HPV-specific testing. This reliance on p16 positivity may introduce bias due to possible discordance between p16 expression and actual HPV infection status.^18^

Experimental HPV-targeted therapeutics and ICI combination studies were excluded from our data analysis due to their unknown impacts on survival (Table 2). However, survival outcomes in these trials were consistently higher than the historical benchmark based on KEYNOTE-048. This apparent improvement may arise from the fact that the experimental groups in these more recent trials comprised only HPV+ patients, whereas the historical benchmark control group included both HPV+ and HPV− patients.

**Table 2.** Clinical trial summary of novel HPV-targeted drugs in HPV + HNSCC.

	Drug (Trial name)/ClinicalTrials.gov ID	Study design	Intervention	*N*	mOS (m)	ORR (%)	mPFS (m)
**First-line**	PDS0101 (VERSATILE-002)/NCT04260126^19^	Single-arm Phase II	PDS0101 + pembrolizumab	53	30.0	35.8	6.3
	HB-200/NCT04180215^20^	Single-arm Phase II	HB-200 + pembrolizumab	35	NR	37.1	NR
	CUE-101/NCT03978689^21^	Multi-cohort Phase I	CUE-101 + pembrolizumab	24	NR *12-month OS 95.5%	46.0	5.8
	ISA101b (OpcemISA)/NCT03669718^22^	Randomized Phase II	ISA101b + cemiplimab	98	15.8	25.3	NR
			Placebo + cemiplimab	100	26.9	22.9	NR
	BNT113 (AHEAD-MERIT) NCT04534205	Randomized Phase II	BNT113 + pembrolizumab	15	22.6	33.3	6.0
**Second or later-line**	CUE-101/NCT03978689^21^	Multi-cohort Phase I	CUE-101	20	20.8	5.0	NR
	ISA101b/NCT04398524^23^	Single-arm Phase II	ISA101b + cemiplimab	26	11.6	15.4	3.9
	MEDI0457/NCT03162224^24^	Phase Ib/IIa	MEDI0457 + durvalumab	35	29.2	25.7	3.8

Abbreviations: HNSCC, head and neck squamous cell carcinoma; HPV, human papillomavirus; N, number; NR, not reached or not reported; mOS, median overall survival; mPFS, median progression-free survival; ORR, objective response rate.

We propose that all trials in R/M HNSCC should report HPV-specific outcomes, especially given that HPV status is frequently used as a stratifying covariate in clinical trials that enroll both HPV+ and HPV− populations. This approach would make post hoc comparisons between these 2 groups more meaningful. In single-arm trials that use historical controls to define success thresholds, investigators should ensure that historical controls have a similar composition of HPV+ and HPV− patients to the study population or establish separate thresholds for what constitutes a positive result in the HPV+ and HPV− subgroups.

## Conclusion

The prognostic outcomes of R/M HNSCC differ significantly between HPV+ and HPV− populations. Our findings indicate that establishing specific outcome benchmarks for the HPV+ and HPV− cohorts is essential for improving treatment strategies and clinical trial designs. These results, derived from available clinical trial data, reflect the current state of therapeutic outcomes in HPV+ and HPV− R/M HNSCC treatment. Furthermore, research and validation in larger, stratified cohorts are needed to establish and confirm new outcome consensus benchmarks specific to HPV+ and HPV− populations. This will help optimize therapeutic development, improve the accuracy of clinical trial data interpretation, and ultimately lead to better patient outcomes.

## Supplementary Material

oyaf043_suppl_Supplementary_Figures_1

## Data Availability

The data underlying this article will be shared on reasonable request to the corresponding author.
