# Supplementary material for: Outcomes for recurrent or metastatic head and neck cancer by HPV status: a systematic review and meta-analysis
Source: Oncologist. 2025 Apr 11;30(4):oyaf043. doi: 10.1093/oncolo/oyaf043 (PMC11986418; doi:10.1093/oncolo/oyaf043)

**Supplementary Material**

**Detailed synthesis methods**

When summary statistics, such as standard deviation (SD), were missing or not directly reported in the included studies, we employed several methods to estimate the standard deviation from other available statistics, such as confidence intervals (CI). The following approaches were used:

(1) When the 95% CI was provided: SD was estimated by dividing the difference between the upper and lower CI by 3.92.

(2) When upper CI was reported as not reached (NR): The upper CI was approximated using a specific method that involves assuming symmetry in the log scale and using the given lower CI to estimate the upper CI. This method assumes that the log-transformed survival times follow a normal distribution and that the 95% CI is symmetric around the median or mean on the log scale. The steps are as follows:

The steps are as follows:

1. Identify the median outcome value (M)

2. Calculate the distance (D) between the median outcome value (M) and the lower CI (L) using the formula: D = M − L

3. Estimate the upper CI (U) by adding the distance (D) to the median outcome value (M). This can be expressed as: U = M + D or U = M + (M − L) = 2M − L

(3) When both the upper and lower CIs were not given: When both upper and lower CIs are not provided, the SD is approximated under the assumption that survival times follow an exponential distribution. This assumption allows us to use the relationship between the median and the mean in an exponential distribution. The mean and the SD in an exponential distribution are equal, and the mean can be approximated from the median using the formula: Mean = M x log(2), where log(2) ≈ 0.693. Consequently, the SD can be approximated as: SD ≈ M x 0.693. This approximation provides a straightforward method to estimate the SD when only the median outcome value is available.

**Limitations of synthesis methods**

When the upper CI was reported as not reached (NR), it was approximated using a method involving the given lower CI. This method assumes that the log-transformed survival times follow a normal distribution and that the 95% CI is symmetric around the median or mean on the log scale. However, this method has several limitations: (1) It assumes that the log-transformed survival times follow a normal distribution, which may not always be true. (2) The accuracy of the estimated upper CI heavily depends on the accuracy of the median and lower CI values. (3) It assumes a constant hazard function over time, which may oversimplify survival dynamics. When both the upper and lower CIs were not provided, the SD was approximated under the assumption that survival times follow an exponential distribution. While this simplifies the analysis, it implies a constant hazard rate over time, potentially oversimplifying survival dynamics.

**Supplementary Figure 1.** Risk of bias graph for included studies


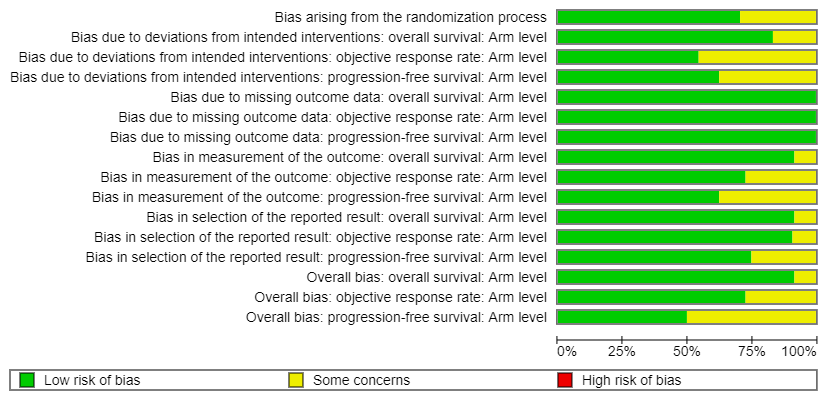

Supplement: oyaf043_suppl_Supplementary_Figures_1 [file oyaf043_suppl_supplementary_figures_1.docx]
